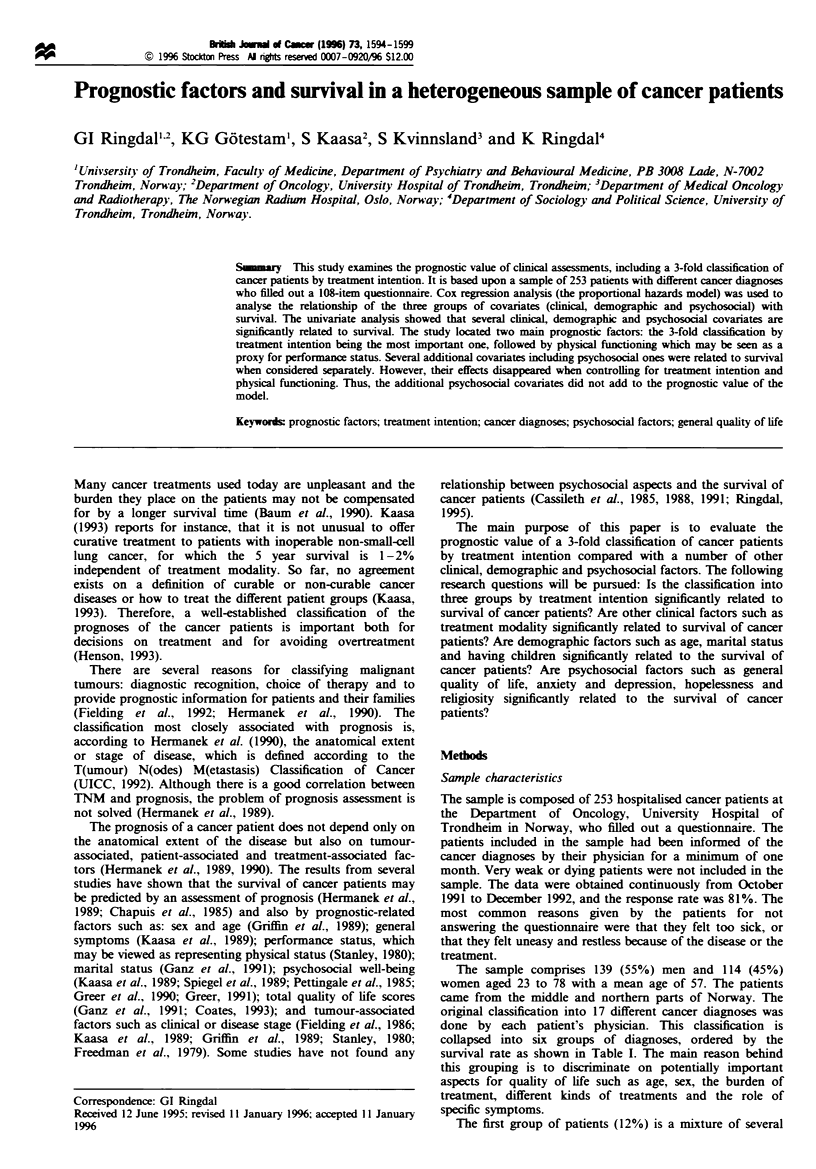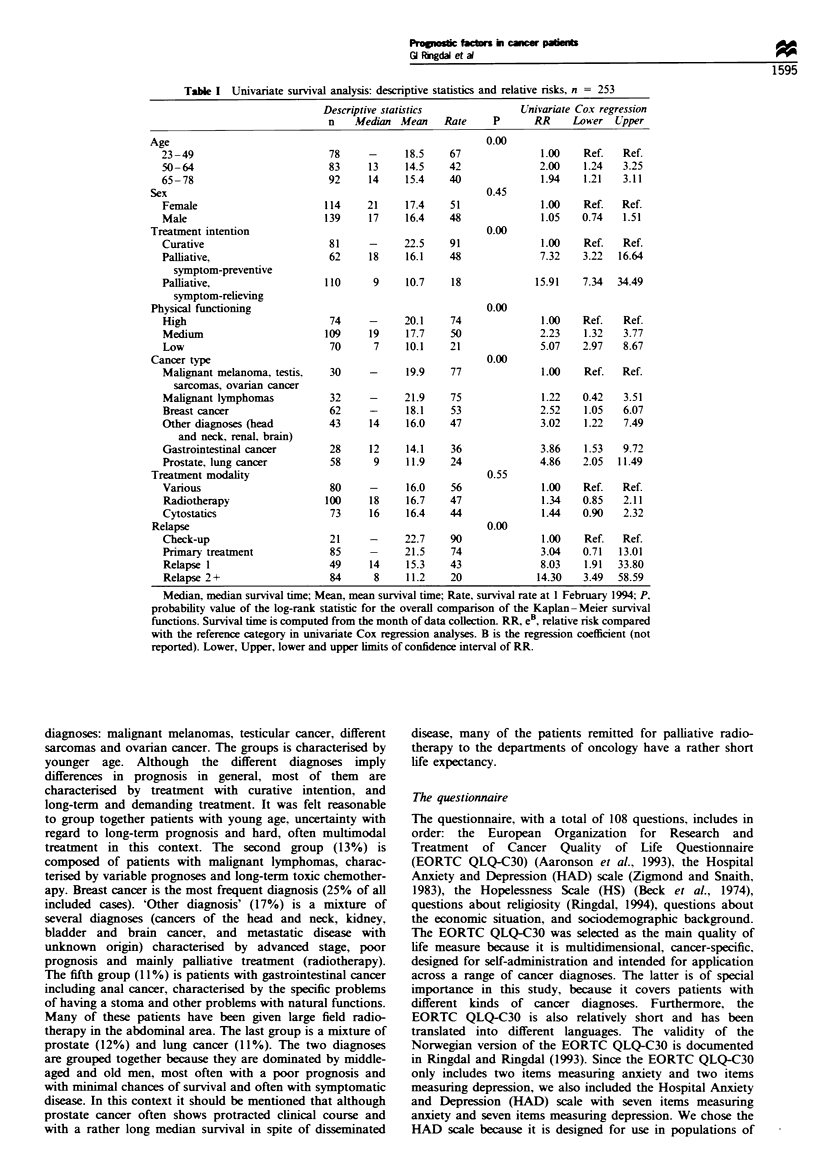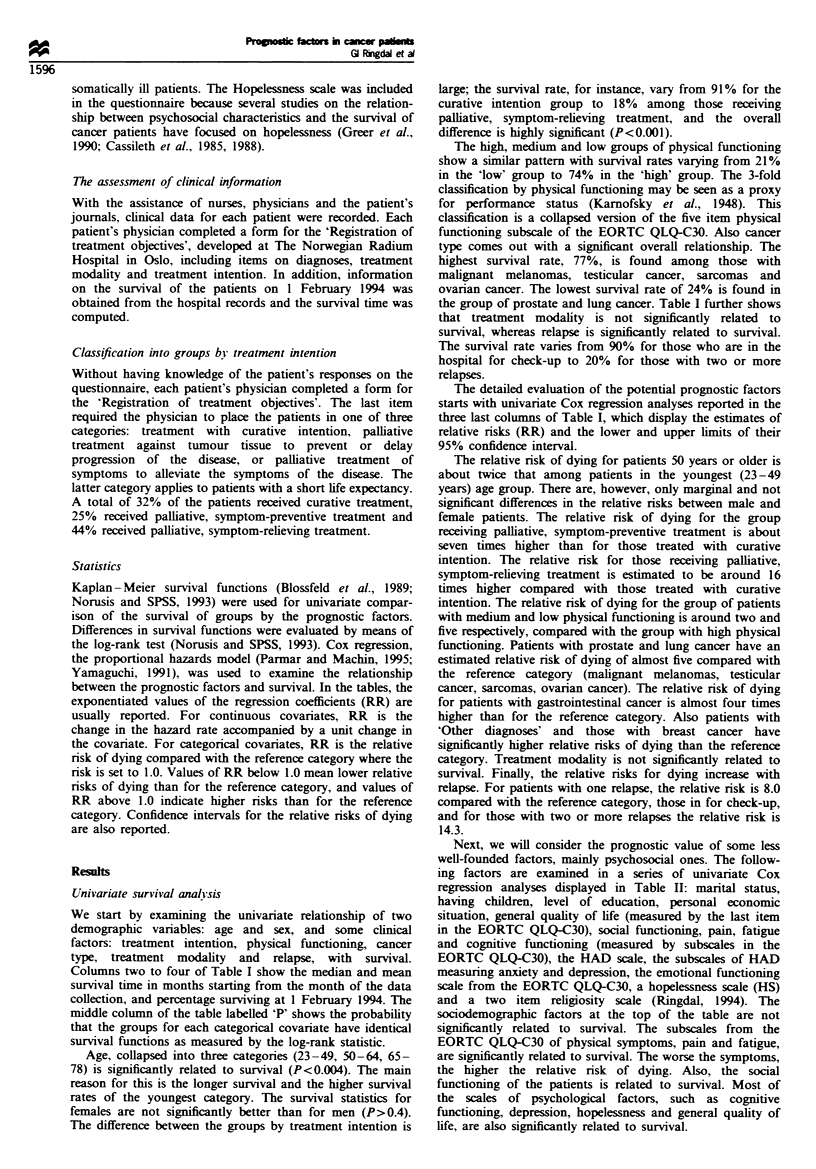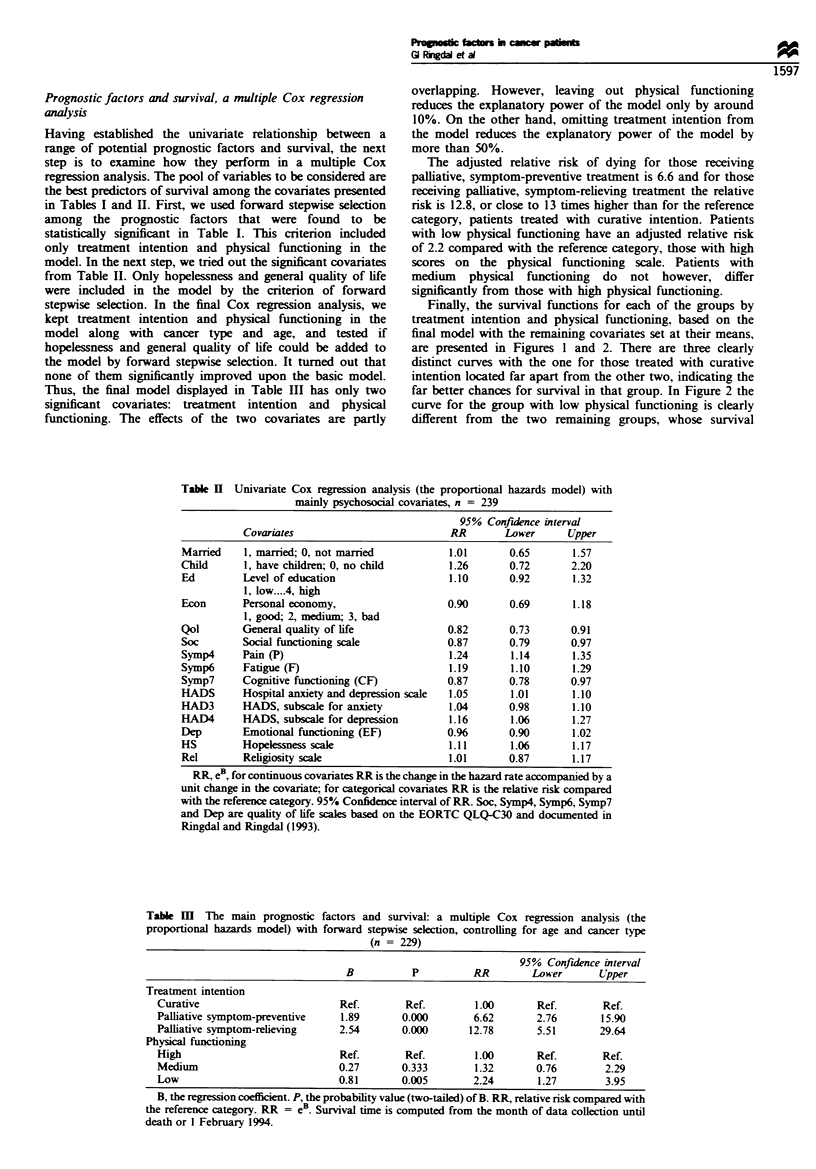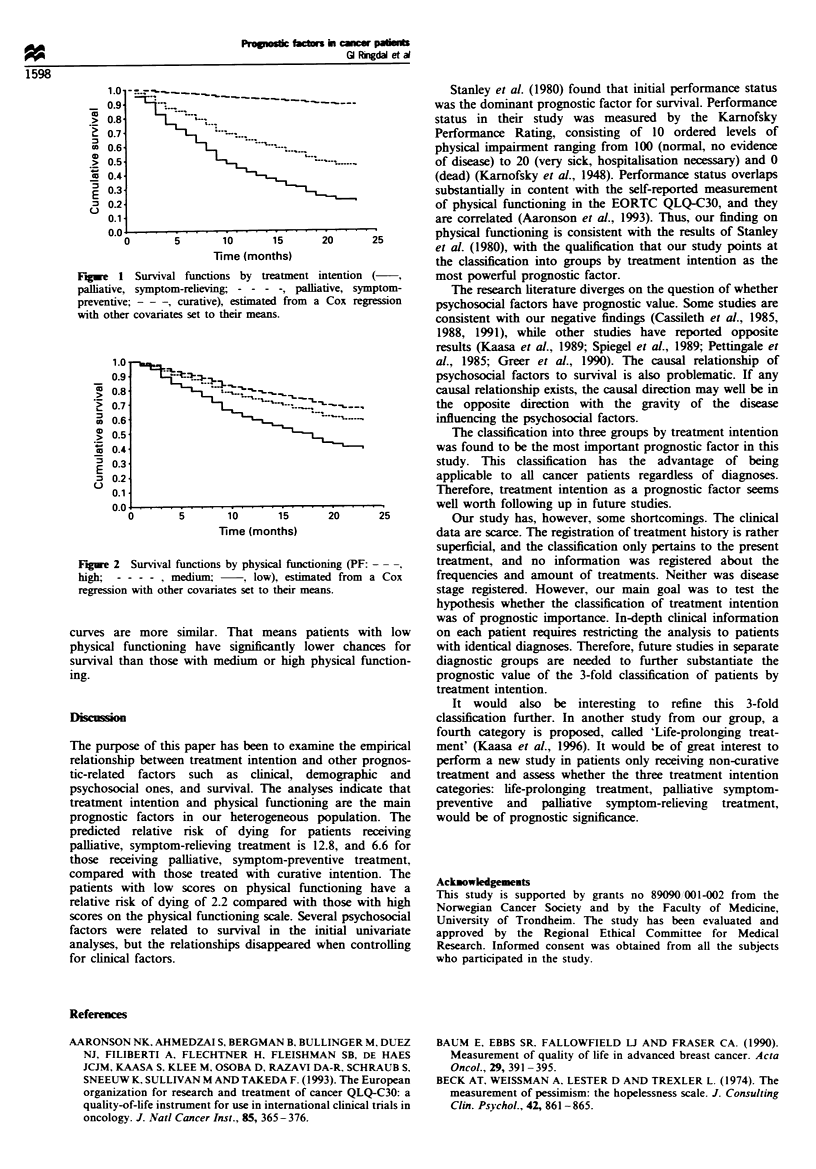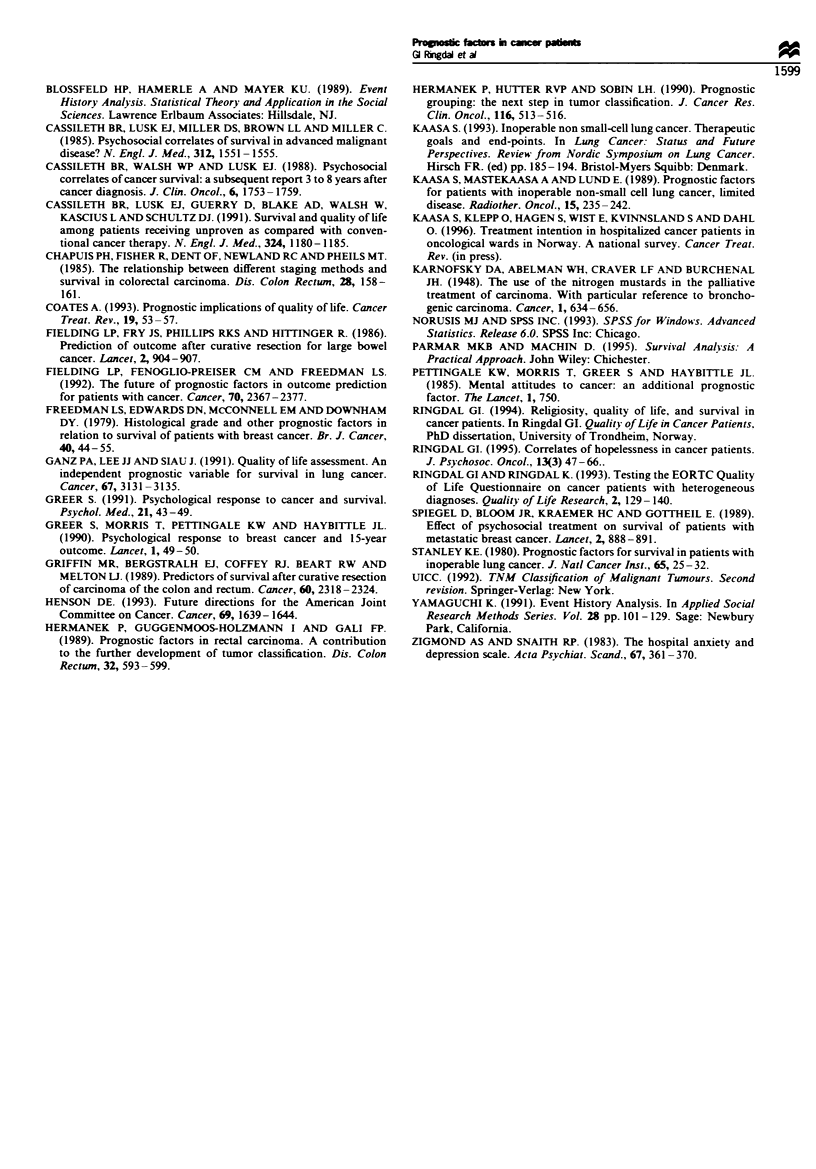# Prognostic factors and survival in a heterogeneous sample of cancer patients.

**DOI:** 10.1038/bjc.1996.300

**Published:** 1996-06

**Authors:** G. I. Ringdal, K. G. Götestam, S. Kaasa, S. Kvinnsland, K. Ringdal

**Affiliations:** University of Trondheim, Faculty of Medicine, Department of Psychiatry and Behavioural Medicine, Norway.

## Abstract

This study examines the prognostic value of clinical assessments, including a 3-fold classification of cancer patients by treatment intention. It is based upon a sample of 253 patients with different cancer diagnoses who filled out a 108-item questionnaire. Cox regression analysis (the proportional hazards model) was used to analyse the relationship of the three groups of covariates (clinical, demographic and psychosocial) with survival. The univariate analysis showed that several clinical, demographic and psychosocial covariates are significantly related to survival. The study located two main prognostic factors: the 3-fold classification by treatment intention being the most important one, followed by physical functioning which may be seen as a proxy for performance status. Several additional covariates including psychosocial ones were related to survival when considered separately. However, their effects disappeared when controlling for treatment intention and physical functioning. Thus, the additional psychosocial covariates did no add to the prognostic value of the model.


British Journal of Cancer (1996) 73, 1594-1599

© 1996 Stockton Press All rights reserved 0007-0920/96 $12.00

### Prognostic factors and survival in a heterogeneous sample of cancer patients

GI Ringdal[1,2], KG Götestam[1], S Kaasa[2], S Kvinnsland[3] and K Ringdal[4]

*[1]Univsersity of Trondheim, Faculty of Medicine, Department of Psychiatry and Behavioural Medicine, PB 3008 Lade, N-7002 Trondheim, Norway; [2]Department of Oncology, University Hospital of Trondheim, Trondheim; [3]Department of Medical Oncology and Radiotherapy, The Norwegian Radium Hospital, Oslo, Norway; [4]Department of Sociology and Political Science, University of Trondheim, Trondheim, Norway.*

**Summary** This study examines the prognostic value of clinical assessments, including a 3-fold classification of cancer patients by treatment intention. It is based upon a sample of 253 patients with different cancer diagnoses who filled out a 108-item questionnaire. Cox regression analysis (the proportional hazards model) was used to analyse the relationship of the three groups of covariates (clinical, demographic and psychosocial) with survival. The univariate analysis showed that several clinical, demographic and psychosocial covariates are significantly related to survival. The study located two main prognostic factors: the 3-fold classification by treatment intention being the most important one, followed by physical functioning which may be seen as a proxy for performance status. Several additional covariates including psychosocial ones were related to survival when considered separately. However, their effects disappeared when controlling for treatment intention and physical functioning. Thus, the additional psychosocial covariates did not add to the prognostic value of the model.

**Keywords:** prognostic factors; treatment intention; cancer diagnoses; psychosocial factors; general quality of life

Many cancer treatments used today are unpleasant and the burden they place on the patients may not be compensated for by a longer survival time (Baum *et al.*, 1990). Kaasa (1993) reports for instance, that it is not unusual to offer curative treatment to patients with inoperable non-small-cell lung cancer, for which the 5 year survival is 1-2% independent of treatment modality. So far, no agreement exists on a definition of curable or non-curable cancer diseases or how to treat the different patient groups (Kaasa, 1993). Therefore, a well-established classification of the prognoses of the cancer patients is important both for decisions on treatment and for avoiding overtreatment (Henson, 1993).

There are several reasons for classifying malignant tumours: diagnostic recognition, choice of therapy and to provide prognostic information for patients and their families (Fielding *et al.*, 1992; Hermanek *et al.*, 1990). The classification most closely associated with prognosis is, according to Hermanek *et al.* (1990), the anatomical extent or stage of disease, which is defined according to the T(umour) N(odes) M(etastasis) Classification of Cancer (UICC, 1992). Although there is a good correlation between TNM and prognosis, the problem of prognosis assessment is not solved (Hermanek *et al.*, 1989).

The prognosis of a cancer patient does not depend only on the anatomical extent of the disease but also on tumour-associated, patient-associated and treatment-associated factors (Hermanek *et al.*, 1989, 1990). The results from several studies have shown that the survival of cancer patients may be predicted by an assessment of prognosis (Hermanek *et al.*, 1989; Chapuis *et al.*, 1985) and also by prognostic-related factors such as: sex and age (Griffin *et al.*, 1989); general symptoms (Kaasa *et al.*, 1989); performance status, which may be viewed as representing physical status (Stanley, 1980); marital status (Ganz *et al.*, 1991); psychosocial well-being (Kaasa *et al.*, 1989; Spiegel *et al.*, 1989; Pettingale *et al.*, 1985; Greer *et al.*, 1990; Greer, 1991); total quality of life scores (Ganz *et al.*, 1991; Coates, 1993); and tumour-associated factors such as clinical or disease stage (Fielding *et al.*, 1986; Kaasa *et al.*, 1989; Griffin *et al.*, 1989; Stanley, 1980; Freedman *et al.*, 1979). Some studies have not found any relationship between psychosocial aspects and the survival of cancer patients (Cassileth *et al.*, 1985, 1988, 1991; Ringdal, 1995).

The main purpose of this paper is to evaluate the prognostic value of a 3-fold classification of cancer patients by treatment intention compared with a number of other clinical, demographic and psychosocial factors. The following research questions will be pursued: Is the classification into three groups by treatment intention significantly related to survival of cancer patients? Are other clinical factors such as treatment modality significantly related to survival of cancer patients? Are demographic factors such as age, marital status and having children significantly related to the survival of cancer patients? Are psychosocial factors such as general quality of life, anxiety and depression, hopelessness and religiosity significantly related to the survival of cancer patients?

### Methods

### *Sample characteristics*

The sample is composed of 253 hospitalised cancer patients at the Department of Oncology, University Hospital of Trondheim in Norway, who filled out a questionnaire. The patients included in the sample had been informed of the cancer diagnoses by their physician for a minimum of one month. Very weak or dying patients were not included in the sample. The data were obtained continuously from October 1991 to December 1992, and the response rate was 81%. The most common reasons given by the patients for not answering the questionnaire were that they felt too sick, or that they felt uneasy and restless because of the disease or the treatment.

The sample comprises 139 (55%) men and 114 (45%) women aged 23 to 78 with a mean age of 57. The patients came from the middle and northern parts of Norway. The original classification into 17 different cancer diagnoses was done by each patient's physician. This classification is collapsed into six groups of diagnoses, ordered by the survival rate as shown in Table I. The main reason behind this grouping is to discriminate on potentially important aspects for quality of life such as age, sex, the burden of treatment, different kinds of treatments and the role of specific symptoms.

The first group of patients (12%) is a mixture of several

Correspondence: GI Ringdal
Received 12 June 1995; revised 11 January 1996; accepted 11 January 1996

**Table I** Univariate survival analysis: descriptive statistics and relative risks, *n* = 253

| | *Descriptive statistics* n | *Median* | *Mean* | *Rate* | P | *Univariate Cox regression* *RR* | *Lower* | *Upper* |
|---|---|---|---|---|---|---|---|---|
| Age | | | | | 0.00 | | | |
| 23–49 | 78 | – | 18.5 | 67 | | 1.00 | Ref. | Ref. |
| 50–64 | 83 | 13 | 14.5 | 42 | | 2.00 | 1.24 | 3.25 |
| 65–78 | 92 | 14 | 15.4 | 40 | | 1.94 | 1.21 | 3.11 |
| Sex | | | | | 0.45 | | | |
| Female | 114 | 21 | 17.4 | 51 | | 1.00 | Ref. | Ref. |
| Male | 139 | 17 | 16.4 | 48 | | 1.05 | 0.74 | 1.51 |
| Treatment intention | | | | | 0.00 | | | |
| Curative | 81 | – | 22.5 | 91 | | 1.00 | Ref. | Ref. |
| Palliative, symptom-preventive | 62 | 18 | 16.1 | 48 | | 7.32 | 3.22 | 16.64 |
| Palliative, symptom-relieving | 110 | 9 | 10.7 | 18 | | 15.91 | 7.34 | 34.49 |
| Physical functioning | | | | | 0.00 | | | |
| High | 74 | – | 20.1 | 74 | | 1.00 | Ref. | Ref. |
| Medium | 109 | 19 | 17.7 | 50 | | 2.23 | 1.32 | 3.77 |
| Low | 70 | 7 | 10.1 | 21 | | 5.07 | 2.97 | 8.67 |
| Cancer type | | | | | 0.00 | | | |
| Malignant melanoma, testis, sarcomas, ovarian cancer | 30 | – | 19.9 | 77 | | 1.00 | Ref. | Ref. |
| Malignant lymphomas | 32 | – | 21.9 | 75 | | 1.22 | 0.42 | 3.51 |
| Breast cancer | 62 | – | 18.1 | 53 | | 2.52 | 1.05 | 6.07 |
| Other diagnoses (head and neck, renal, brain) | 43 | 14 | 16.0 | 47 | | 3.02 | 1.22 | 7.49 |
| Gastrointestinal cancer | 28 | 12 | 14.1 | 36 | | 3.86 | 1.53 | 9.72 |
| Prostate, lung cancer | 58 | 9 | 11.9 | 24 | | 4.86 | 2.05 | 11.49 |
| Treatment modality | | | | | 0.55 | | | |
| Various | 80 | – | 16.0 | 56 | | 1.00 | Ref. | Ref. |
| Radiotherapy | 100 | 18 | 16.7 | 47 | | 1.34 | 0.85 | 2.11 |
| Cytostatics | 73 | 16 | 16.4 | 44 | | 1.44 | 0.90 | 2.32 |
| Relapse | | | | | 0.00 | | | |
| Check-up | 21 | – | 22.7 | 90 | | 1.00 | Ref. | Ref. |
| Primary treatment | 85 | – | 21.5 | 74 | | 3.04 | 0.71 | 13.01 |
| Relapse 1 | 49 | 14 | 15.3 | 43 | | 8.03 | 1.91 | 33.80 |
| Relapse 2+ | 84 | 8 | 11.2 | 20 | | 14.30 | 3.49 | 58.59 |

Median, median survival time; Mean, mean survival time; Rate, survival rate at 1 February 1994; *P*, probability value of the log-rank statistic for the overall comparison of the Kaplan–Meier survival functions. Survival time is computed from the month of data collection. RR, $e^B$, relative risk compared with the reference category in univariate Cox regression analyses. B is the regression coefficient (not reported). Lower, Upper, lower and upper limits of confidence interval of RR.

diagnoses: malignant melanomas, testicular cancer, different sarcomas and ovarian cancer. The groups is characterised by younger age. Although the different diagnoses imply differences in prognosis in general, most of them are characterised by treatment with curative intention, and long-term and demanding treatment. It was felt reasonable to group together patients with young age, uncertainty with regard to long-term prognosis and hard, often multimodal treatment in this context. The second group (13%) is composed of patients with malignant lymphomas, characterised by variable prognoses and long-term toxic chemotherapy. Breast cancer is the most frequent diagnosis (25% of all included cases). 'Other diagnosis' (17%) is a mixture of several diagnoses (cancers of the head and neck, kidney, bladder and brain cancer, and metastatic disease with unknown origin) characterised by advanced stage, poor prognosis and mainly palliative treatment (radiotherapy). The fifth group (11%) is patients with gastrointestinal cancer including anal cancer, characterised by the specific problems of having a stoma and other problems with natural functions. Many of these patients have been given large field radiotherapy in the abdominal area. The last group is a mixture of prostate (12%) and lung cancer (11%). The two diagnoses are grouped together because they are dominated by middle-aged and old men, most often with a poor prognosis and with minimal chances of survival and often with symptomatic disease. In this context it should be mentioned that although prostate cancer often shows protracted clinical course and with a rather long median survival in spite of disseminated disease, many of the patients remitted for palliative radiotherapy to the departments of oncology have a rather short life expectancy.

### *The questionnaire*

The questionnaire, with a total of 108 questions, includes in order: the European Organization for Research and Treatment of Cancer Quality of Life Questionnaire (EORTC QLQ-C30) (Aaronson *et al.*, 1993), the Hospital Anxiety and Depression (HAD) scale (Zigmond and Snaith, 1983), the Hopelessness Scale (HS) (Beck *et al.*, 1974), questions about religiosity (Ringdal, 1994), questions about the economic situation, and sociodemographic background. The EORTC QLQ-C30 was selected as the main quality of life measure because it is multidimensional, cancer-specific, designed for self-administration and intended for application across a range of cancer diagnoses. The latter is of special importance in this study, because it covers patients with different kinds of cancer diagnoses. Furthermore, the EORTC QLQ-C30 is also relatively short and has been translated into different languages. The validity of the Norwegian version of the EORTC QLQ-C30 is documented in Ringdal and Ringdal (1993). Since the EORTC QLQ-C30 only includes two items measuring anxiety and two items measuring depression, we also included the Hospital Anxiety and Depression (HAD) scale with seven items measuring anxiety and seven items measuring depression. We chose the HAD scale because it is designed for use in populations of somatically ill patients. The Hopelessness scale was included in the questionnaire because several studies on the relationship between psychosocial characteristics and the survival of cancer patients have focused on hopelessness (Greer *et al*., 1990; Cassileth *et al*., 1985, 1988).

*The assessment of clinical information*

With the assistance of nurses, physicians and the patient's journals, clinical data for each patient were recorded. Each patient's physician completed a form for the 'Registration of treatment objectives', developed at The Norwegian Radium Hospital in Oslo, including items on diagnoses, treatment modality and treatment intention. In addition, information on the survival of the patients on 1 February 1994 was obtained from the hospital records and the survival time was computed.

*Classification into groups by treatment intention*

Without having knowledge of the patient's responses on the questionnaire, each patient's physician completed a form for the 'Registration of treatment objectives'. The last item required the physician to place the patients in one of three categories: treatment with curative intention, palliative treatment against tumour tissue to prevent or delay progression of the disease, or palliative treatment of symptoms to alleviate the symptoms of the disease. The latter category applies to patients with a short life expectancy. A total of 32% of the patients received curative treatment, 25% received palliative, symptom-preventive treatment and 44% received palliative, symptom-relieving treatment.

*Statistics*

Kaplan–Meier survival functions (Blossfeld *et al*., 1989; Norusis and SPSS, 1993) were used for univariate comparison of the survival of groups by the prognostic factors. Differences in survival functions were evaluated by means of the log-rank test (Norusis and SPSS, 1993). Cox regression, the proportional hazards model (Parmar and Machin, 1995; Yamaguchi, 1991), was used to examine the relationship between the prognostic factors and survival. In the tables, the exponentiated values of the regression coefficients (RR) are usually reported. For continuous covariates, RR is the change in the hazard rate accompanied by a unit change in the covariate. For categorical covariates, RR is the relative risk of dying compared with the reference category where the risk is set to 1.0. Values of RR below 1.0 mean lower relative risks of dying than for the reference category, and values of RR above 1.0 indicate higher risks than for the reference category. Confidence intervals for the relative risks of dying are also reported.

### Results

*Univariate survival analysis*

We start by examining the univariate relationship of two demographic variables: age and sex, and some clinical factors: treatment intention, physical functioning, cancer type, treatment modality and relapse, with survival. Columns two to four of Table I show the median and mean survival time in months starting from the month of the data collection, and percentage surviving at 1 February 1994. The middle column of the table labelled 'P' shows the probability that the groups for each categorical covariate have identical survival functions as measured by the log-rank statistic.

Age, collapsed into three categories (23–49, 50–64, 65–78) is significantly related to survival ($P<0.004$). The main reason for this is the longer survival and the higher survival rates of the youngest category. The survival statistics for females are not significantly better than for men ($P>0.4$). The difference between the groups by treatment intention is large; the survival rate, for instance, vary from 91% for the curative intention group to 18% among those receiving palliative, symptom-relieving treatment, and the overall difference is highly significant ($P<0.001$).

The high, medium and low groups of physical functioning show a similar pattern with survival rates varying from 21% in the 'low' group to 74% in the 'high' group. The 3-fold classification by physical functioning may be seen as a proxy for performance status (Karnofsky *et al*., 1948). This classification is a collapsed version of the five item physical functioning subscale of the EORTC QLQ-C30. Also cancer type comes out with a significant overall relationship. The highest survival rate, 77%, is found among those with malignant melanomas, testicular cancer, sarcomas and ovarian cancer. The lowest survival rate of 24% is found in the group of prostate and lung cancer. Table I further shows that treatment modality is not significantly related to survival, whereas relapse is significantly related to survival. The survival rate varies from 90% for those who are in the hospital for check-up to 20% for those with two or more relapses.

The detailed evaluation of the potential prognostic factors starts with univariate Cox regression analyses reported in the three last columns of Table I, which display the estimates of relative risks (RR) and the lower and upper limits of their 95% confidence interval.

The relative risk of dying for patients 50 years or older is about twice that among patients in the youngest (23–49 years) age group. There are, however, only marginal and not significant differences in the relative risks between male and female patients. The relative risk of dying for the group receiving palliative, symptom-preventive treatment is about seven times higher than for those treated with curative intention. The relative risk for those receiving palliative, symptom-relieving treatment is estimated to be around 16 times higher compared with those treated with curative intention. The relative risk of dying for the group of patients with medium and low physical functioning is around two and five respectively, compared with the group with high physical functioning. Patients with prostate and lung cancer have an estimated relative risk of dying of almost five compared with the reference category (malignant melanomas, testicular cancer, sarcomas, ovarian cancer). The relative risk of dying for patients with gastrointestinal cancer is almost four times higher than for the reference category. Also patients with 'Other diagnoses' and those with breast cancer have significantly higher relative risks of dying than the reference category. Treatment modality is not significantly related to survival. Finally, the relative risks for dying increase with relapse. For patients with one relapse, the relative risk is 8.0 compared with the reference category, those in for check-up, and for those with two or more relapses the relative risk is 14.3.

Next, we will consider the prognostic value of some less well-founded factors, mainly psychosocial ones. The following factors are examined in a series of univariate Cox regression analyses displayed in Table II: marital status, having children, level of education, personal economic situation, general quality of life (measured by the last item in the EORTC QLQ-C30), social functioning, pain, fatigue and cognitive functioning (measured by subscales in the EORTC QLQ-C30), the HAD scale, the subscales of HAD measuring anxiety and depression, the emotional functioning scale from the EORTC QLQ-C30, a hopelessness scale (HS) and a two item religiosity scale (Ringdal, 1994). The sociodemographic factors at the top of the table are not significantly related to survival. The subscales from the EORTC QLQ-C30 of physical symptoms, pain and fatigue, are significantly related to survival. The worse the symptoms, the higher the relative risk of dying. Also, the social functioning of the patients is related to survival. Most of the scales of psychological factors, such as cognitive functioning, depression, hopelessness and general quality of life, are also significantly related to survival.

### *Prognostic factors and survival, a multiple Cox regression analysis*

Having established the univariate relationship between a range of potential prognostic factors and survival, the next step is to examine how they perform in a multiple Cox regression analysis. The pool of variables to be considered are the best predictors of survival among the covariates presented in Tables I and II. First, we used forward stepwise selection among the prognostic factors that were found to be statistically significant in Table I. This criterion included only treatment intention and physical functioning in the model. In the next step, we tried out the significant covariates from Table II. Only hopelessness and general quality of life were included in the model by the criterion of forward stepwise selection. In the final Cox regression analysis, we kept treatment intention and physical functioning in the model along with cancer type and age, and tested if hopelessness and general quality of life could be added to the model by forward stepwise selection. It turned out that none of them significantly improved upon the basic model. Thus, the final model displayed in Table III has only two significant covariates: treatment intention and physical functioning. The effects of the two covariates are partly overlapping. However, leaving out physical functioning reduces the explanatory power of the model only by around 10%. On the other hand, omitting treatment intention from the model reduces the explanatory power of the model by more than 50%.

The adjusted relative risk of dying for those receiving palliative, symptom-preventive treatment is 6.6 and for those receiving palliative, symptom-relieving treatment the relative risk is 12.8, or close to 13 times higher than for the reference category, patients treated with curative intention. Patients with low physical functioning have an adjusted relative risk of 2.2 compared with the reference category, those with high scores on the physical functioning scale. Patients with medium physical functioning do not however, differ significantly from those with high physical functioning.

Finally, the survival functions for each of the groups by treatment intention and physical functioning, based on the final model with the remaining covariates set at their means, are presented in Figures 1 and 2. There are three clearly distinct curves with the one for those treated with curative intention located far apart from the other two, indicating the far better chances for survival in that group. In Figure 2 the curve for the group with low physical functioning is clearly different from the two remaining groups, whose survival

**Table II** Univariate Cox regression analysis (the proportional hazards model) with mainly psychosocial covariates, $n = 239$

| | *Covariates* | *RR* | *95% Confidence interval Lower* | *Upper* |
|---|---|---|---|---|
| Married | 1, married; 0, not married | 1.01 | 0.65 | 1.57 |
| Child | 1, have children; 0, no child | 1.26 | 0.72 | 2.20 |
| Ed | Level of education 1, low....4, high | 1.10 | 0.92 | 1.32 |
| Econ | Personal economy, 1, good; 2, medium; 3, bad | 0.90 | 0.69 | 1.18 |
| Qol | General quality of life | 0.82 | 0.73 | 0.91 |
| Soc | Social functioning scale | 0.87 | 0.79 | 0.97 |
| Symp4 | Pain (P) | 1.24 | 1.14 | 1.35 |
| Symp6 | Fatigue (F) | 1.19 | 1.10 | 1.29 |
| Symp7 | Cognitive functioning (CF) | 0.87 | 0.78 | 0.97 |
| HADS | Hospital anxiety and depression scale | 1.05 | 1.01 | 1.10 |
| HAD3 | HADS, subscale for anxiety | 1.04 | 0.98 | 1.10 |
| HAD4 | HADS, subscale for depression | 1.16 | 1.06 | 1.27 |
| Dep | Emotional functioning (EF) | 0.96 | 0.90 | 1.02 |
| HS | Hopelessness scale | 1.11 | 1.06 | 1.17 |
| Rel | Religiosity scale | 1.01 | 0.87 | 1.17 |

RR, $e^B$, for continuous covariates RR is the change in the hazard rate accompanied by a unit change in the covariate; for categorical covariates RR is the relative risk compared with the reference category. 95% Confidence interval of RR. Soc, Symp4, Symp6, Symp7 and Dep are quality of life scales based on the EORTC QLQ-C30 and documented in Ringdal and Ringdal (1993).

**Table III** The main prognostic factors and survival: a multiple Cox regression analysis (the proportional hazards model) with forward stepwise selection, controlling for age and cancer type ($n = 229$)

| | *B* | P | *RR* | *95% Confidence interval Lower* | *Upper* |
|---|---|---|---|---|---|
| Treatment intention | | | | | |
| Curative | Ref. | Ref. | 1.00 | Ref. | Ref. |
| Palliative symptom-preventive | 1.89 | 0.000 | 6.62 | 2.76 | 15.90 |
| Palliative symptom-relieving | 2.54 | 0.000 | 12.78 | 5.51 | 29.64 |
| Physical functioning | | | | | |
| High | Ref. | Ref. | 1.00 | Ref. | Ref. |
| Medium | 0.27 | 0.333 | 1.32 | 0.76 | 2.29 |
| Low | 0.81 | 0.005 | 2.24 | 1.27 | 3.95 |

B, the regression coefficient. *P*, the probability value (two-tailed) of B. RR, relative risk compared with the reference category. RR = $e^B$. Survival time is computed from the month of data collection until death or 1 February 1994.

**Figure 1** Survival functions by treatment intention (——, palliative, symptom-relieving; - - - -, palliative, symptom-preventive; – – –, curative), estimated from a Cox regression with other covariates set to their means.

**Figure 2** Survival functions by physical functioning (PF: – – –, high; - - - - , medium; ——, low), estimated from a Cox regression with other covariates set to their means.

curves are more similar. That means patients with low physical functioning have significantly lower chances for survival than those with medium or high physical functioning.

### Discussion

The purpose of this paper has been to examine the empirical relationship between treatment intention and other prognostic-related factors such as clinical, demographic and psychosocial ones, and survival. The analyses indicate that treatment intention and physical functioning are the main prognostic factors in our heterogeneous population. The predicted relative risk of dying for patients receiving palliative, symptom-relieving treatment is 12.8, and 6.6 for those receiving palliative, symptom-preventive treatment, compared with those treated with curative intention. The patients with low scores on physical functioning have a relative risk of dying of 2.2 compared with those with high scores on the physical functioning scale. Several psychosocial factors were related to survival in the initial univariate analyses, but the relationships disappeared when controlling for clinical factors.

Stanley *et al.* (1980) found that initial performance status was the dominant prognostic factor for survival. Performance status in their study was measured by the Karnofsky Performance Rating, consisting of 10 ordered levels of physical impairment ranging from 100 (normal, no evidence of disease) to 20 (very sick, hospitalisation necessary) and 0 (dead) (Karnofsky *et al.*, 1948). Performance status overlaps substantially in content with the self-reported measurement of physical functioning in the EORTC QLQ-C30, and they are correlated (Aaronson *et al.*, 1993). Thus, our finding on physical functioning is consistent with the results of Stanley *et al.* (1980), with the qualification that our study points at the classification into groups by treatment intention as the most powerful prognostic factor.

The research literature diverges on the question of whether psychosocial factors have prognostic value. Some studies are consistent with our negative findings (Cassileth *et al.*, 1985, 1988, 1991), while other studies have reported opposite results (Kaasa *et al.*, 1989; Spiegel *et al.*, 1989; Pettingale *et al.*, 1985; Greer *et al.*, 1990). The causal relationship of psychosocial factors to survival is also problematic. If any causal relationship exists, the causal direction may well be in the opposite direction with the gravity of the disease influencing the psychosocial factors.

The classification into three groups by treatment intention was found to be the most important prognostic factor in this study. This classification has the advantage of being applicable to all cancer patients regardless of diagnoses. Therefore, treatment intention as a prognostic factor seems well worth following up in future studies.

Our study has, however, some shortcomings. The clinical data are scarce. The registration of treatment history is rather superficial, and the classification only pertains to the present treatment, and no information was registered about the frequencies and amount of treatments. Neither was disease stage registered. However, our main goal was to test the hypothesis whether the classification of treatment intention was of prognostic importance. In-depth clinical information on each patient requires restricting the analysis to patients with identical diagnoses. Therefore, future studies in separate diagnostic groups are needed to further substantiate the prognostic value of the 3-fold classification of patients by treatment intention.

It would also be interesting to refine this 3-fold classification further. In another study from our group, a fourth category is proposed, called 'Life-prolonging treatment' (Kaasa *et al.*, 1996). It would be of great interest to perform a new study in patients only receiving non-curative treatment and assess whether the three treatment intention categories: life-prolonging treatment, palliative symptom-preventive and palliative symptom-relieving treatment, would be of prognostic significance.

**Acknowledgements**
This study is supported by grants no 89090/001-002 from the Norwegian Cancer Society and by the Faculty of Medicine, University of Trondheim. The study has been evaluated and approved by the Regional Ethical Committee for Medical Research. Informed consent was obtained from all the subjects who participated in the study.

### References

AARONSON NK, AHMEDZAI S, BERGMAN B, BULLINGER M, DUEZ NJ, FILIBERTI A, FLECHTNER H, FLEISHMAN SB, DE HAES JCJM, KAASA S, KLEE M, OSOBA D, RAZAVI DA-R, SCHRAUB S, SNEEUW K, SULLIVAN M AND TAKEDA F. (1993). The European organization for research and treatment of cancer QLQ-C30: a quality-of-life instrument for use in international clinical trials in oncology. *J. Natl Cancer Inst.*, **85**, 365–376.

BAUM E, EBBS SR, FALLOWFIELD LJ AND FRASER CA. (1990). Measurement of quality of life in advanced breast cancer. *Acta Oncol.*, **29**, 391–395.

BECK AT, WEISSMAN A, LESTER D AND TREXLER L. (1974). The measurement of pessimism: the hopelessness scale. *J. Consulting Clin. Psychol.*, **42**, 861–865.

BLOSSFELD HP, HAMERLE A AND MAYER KU. (1989). *Event History Analysis. Statistical Theory and Application in the Social Sciences*. Lawrence Erlbaum Associates: Hillsdale, NJ.

CASSILETH BR, LUSK EJ, MILLER DS, BROWN LL AND MILLER C. (1985). Psychosocial correlates of survival in advanced malignant disease? *N. Engl. J. Med.*, **312**, 1551-1555.

CASSILETH BR, WALSH WP AND LUSK EJ. (1988). Psychosocial correlates of cancer survival: a subsequent report 3 to 8 years after cancer diagnosis. *J. Clin. Oncol.*, **6**, 1753-1759.

CASSILETH BR, LUSK EJ, GUERRY D, BLAKE AD, WALSH W, KASCIUS L AND SCHULTZ DJ. (1991). Survival and quality of life among patients receiving unproven as compared with conventional cancer therapy. *N. Engl. J. Med.*, **324**, 1180-1185.

CHAPUIS PH, FISHER R, DENT OF, NEWLAND RC AND PHEILS MT. (1985). The relationship between different staging methods and survival in colorectal carcinoma. *Dis. Colon Rectum*, **28**, 158-161.

COATES A. (1993). Prognostic implications of quality of life. *Cancer Treat. Rev.*, **19**, 53-57.

FIELDING LP, FRY JS, PHILLIPS RKS AND HITTINGER R. (1986). Prediction of outcome after curative resection for large bowel cancer. *Lancet*, **2**, 904-907.

FIELDING LP, FENOGLIO-PREISER CM AND FREEDMAN LS. (1992). The future of prognostic factors in outcome prediction for patients with cancer. *Cancer*, **70**, 2367-2377.

FREEDMAN LS, EDWARDS DN, MCCONNELL EM AND DOWNHAM DY. (1979). Histological grade and other prognostic factors in relation to survival of patients with breast cancer. *Br. J. Cancer*, **40**, 44-55.

GANZ PA, LEE JJ AND SIAU J. (1991). Quality of life assessment. An independent prognostic variable for survival in lung cancer. *Cancer*, **67**, 3131-3135.

GREER S. (1991). Psychological response to cancer and survival. *Psychol. Med.*, **21**, 43-49.

GREER S, MORRIS T, PETTINGALE KW AND HAYBITTLE JL. (1990). Psychological response to breast cancer and 15-year outcome. *Lancet*, **1**, 49-50.

GRIFFIN MR, BERGSTRALH EJ, COFFEY RJ, BEART RW AND MELTON LJ. (1989). Predictors of survival after curative resection of carcinoma of the colon and rectum. *Cancer*, **60**, 2318-2324.

HENSON DE. (1993). Future directions for the American Joint Committee on Cancer. *Cancer*, **69**, 1639-1644.

HERMANEK P, GUGGENMOOS-HOLZMANN I AND GALI FP. (1989). Prognostic factors in rectal carcinoma. A contribution to the further development of tumor classification. *Dis. Colon Rectum*, **32**, 593-599.

HERMANEK P, HUTTER RVP AND SOBIN LH. (1990). Prognostic grouping: the next step in tumor classification. *J. Cancer Res. Clin. Oncol.*, **116**, 513-516.

KAASA S. (1993). Inoperable non small-cell lung cancer. Therapeutic goals and end-points. In *Lung Cancer: Status and Future Perspectives. Review from Nordic Symposium on Lung Cancer*. Hirsch FR. (ed) pp. 185-194. Bristol-Myers Squibb: Denmark.

KAASA S, MASTEKAASA A AND LUND E. (1989). Prognostic factors for patients with inoperable non-small cell lung cancer, limited disease. *Radiother. Oncol.*, **15**, 235-242.

KAASA S, KLEPP O, HAGEN S, WIST E, KVINNSLAND S AND DAHL O. (1996). Treatment intention in hospitalized cancer patients in oncological wards in Norway. A national survey. *Cancer Treat. Rev.* (in press).

KARNOFSKY DA, ABELMAN WH, CRAVER LF AND BURCHENAL JH. (1948). The use of the nitrogen mustards in the palliative treatment of carcinoma. With particular reference to bronchogenic carcinoma. *Cancer*, **1**, 634-656.

NORUSIS MJ AND SPSS INC. (1993). *SPSS for Windows. Advanced Statistics. Release 6.0*. SPSS Inc: Chicago.

PARMAR MKB AND MACHIN D. (1995). *Survival Analysis: A Practical Approach*. John Wiley: Chichester.

PETTINGALE KW, MORRIS T, GREER S AND HAYBITTLE JL. (1985). Mental attitudes to cancer: an additional prognostic factor. *The Lancet*, **1**, 750.

RINGDAL GI. (1994). Religiosity, quality of life, and survival in cancer patients. In Ringdal GI. *Quality of Life in Cancer Patients*, PhD dissertation, University of Trondheim, Norway.

RINGDAL GI. (1995). Correlates of hopelessness in cancer patients. *J. Psychosoc. Oncol.*, **13(3)** 47-66..

RINGDAL GI AND RINGDAL K. (1993). Testing the EORTC Quality of Life Questionnaire on cancer patients with heterogeneous diagnoses. *Quality of Life Research*, **2**, 129-140.

SPIEGEL D, BLOOM JR, KRAEMER HC AND GOTTHEIL E. (1989). Effect of psychosocial treatment on survival of patients with metastatic breast cancer. *Lancet*, **2**, 888-891.

STANLEY KE. (1980). Prognostic factors for survival in patients with inoperable lung cancer. *J. Natl Cancer Inst.*, **65**, 25-32.

UICC. (1992). *TNM Classification of Malignant Tumours. Second revision*. Springer-Verlag: New York.

YAMAGUCHI K. (1991). Event History Analysis. In *Applied Social Research Methods Series. Vol.* **28** pp. 101-129. Sage: Newbury Park, California.

ZIGMOND AS AND SNAITH RP. (1983). The hospital anxiety and depression scale. *Acta Psychiat. Scand.*, **67**, 361-370.